# Socio-economic disparities in child-to-adolescent growth trajectories in China: Findings from the China Health and Nutrition Survey 1991–2015

**DOI:** 10.1016/j.lanwpc.2022.100399

**Published:** 2022-02-26

**Authors:** Mingyue Gao, Jonathan C.K. Wells, William Johnson, Leah Li

**Affiliations:** a Population, Policy and Practice Research and Teaching Department, University College London Great Ormond Street Institute of Child Health, 30 Guilford Street, London WC1N 1EH, UK; b School of Sport, Exercise and Health Sciences, Loughborough University, Epinal Way, Loughborough, Leicestershire LE11 3TU, UK

**Keywords:** Socio-economic disparities, Height trajectories, BMI trajectories, Chinese children and adolescents, Changes over time, BMI, Body mass index, CHNS, China Health and Nutrition Survey, HAZ, height-for-age z-scores, BAZ, BMI-for-age z-scores, LMICs, low-/middle-income countries, HICs, high-income countries, SEP, socio-economic position

## Abstract

**Backgrounds:**

Socio-economic disparities in growth trajectories of children from low-/middle-income countries are poorly understood, especially those experiencing rapid economic growth. We investigated socio-economic disparities in child growth in recent decades in China.

**Methods:**

Using longitudinal data on 5095 children/adolescents (7–18 years) from the China Health and Nutrition Survey (1991–2015), we estimated mean height and BMI trajectories by socio-economic position (SEP) and sex for cohorts born in 1981–85, 1986–90, 1991–95, 1996–2000, using random-effects models. We estimated differences between high (urbanization index ≥median, household income per capita ≥median, parental education ≥high school, or occupational classes I–IV) and low SEP groups.

**Findings:**

Mean height and BMI trajectories have shifted upwards across cohorts. In all cohorts, growth trajectories for high SEP groups were above those for low SEP groups across SEP indicators. For height, socio-economic differences persisted across cohorts (e.g. 3.8cm and 2.9cm in earliest and latest cohorts by urbanization index for boys at 10 year, and 3.6cm and 3.1cm respectively by household income). For BMI, trends were greater in high than low SEP groups, thus socio-economic differences increased across cohorts (e.g. 0.5 to 0.8kg/m^2^ by urbanization index, 0.4 to 1.1kg/m^2^ by household income for boys at 10 year). Similar trends were found for stunting and overweight/obesity by SEP. There was no association between SEP indicators and thinness.

**Interpretation:**

Socio-economic disparities in physical growth persist among Chinese youth. Short stature was associated with lower SEP, but high BMI with higher SEP. Public health interventions should be tailored by SEP, in order to improve children's growth while reducing overweight/obesity.

**Funding:**

MG is supported by UCL Overseas Research Scholarship and China Scholarship Council for her PhD study. WJ is supported by a UK Medical Research Council (MRC) New Investigator Research Grant (MR/P023347/1) and acknowledges support from the National Institute for Health Research (NIHR) Leicester Biomedical Research Centre, which is a partnership between University Hospitals of Leicester NHS Trust, Loughborough University, and the University of Leicester.

**Research in context**

**Evidence before this study**

Poor growth in height, and high BMI, are two markers of the double burden of malnutrition in children, and both have been linked with poorer cardiometabolic risk in adulthood. We searched for studies, including reviews, of socio-economic disparities in child growth published from Jan 2000 to Sep 2021. We searched in PubMed, Embase and Web of Science with the “AND” logic combination for ‘childhood’, ‘socioeconomic position’ and ‘height/BMI’ concepts with the following standard searching strategy. For ‘**childhood’**, MeSH terms: “child”, “children”, “adolescent”, “adolescence”, free text terms: “child*”, “adolesc*”. For ‘**socioeconomic position**’, MeSH terms: "socioeconomic factors", "social class". Free-text terms: "socio-economic", "socioeconomic", "social inequalit*", "inequalit*", "health disparit*", "socioeconomic gradient*", "social gradient*", "urbanization", "urbaniz*", "urban-rural", "rural-urban", "urban*", "rural*", "educat*", "occupation*", "profession*", "employment*", "income*", "wealth", "poverty", "poor", "deprivation*". For ‘**height/BMI**’, MeSH terms: "obesity", "overweight", "adiposity", "body mass index", "body weight", "body height", "body size". Free-text terms: "obes*", "overweight", "adiposity", "BMI", "body mass index", "body weight", "body height", "stature", "body size". Studies consistently show that children from poor socio-economic backgrounds have shorter stature compared to those from better-off backgrounds. For BMI, associations are in contrasting directions between high-income countries (HICs) and low-/middle-income countries (LMICs), with children from poor socio-economic backgrounds having lower mean BMI in LMICs, but higher BMI in HICs. Furthermore, evidence is inconsistent on secular trends in socio-economic differences in childhood growth trajectories in LMICs. As a rapid developing LMIC, Chinese children have experienced improvements in living standards and changes in physical growth. However, previous evidence from China only focused on area-level indicators of socio-economic position (SEP) such as urban-rural residence, and adopted a cross-sectional analytic design. Thus, socio-economic disparities in age-related height and BMI trajectories, and their secular changes, are not well studied. Such evidence is needed, given the long-term adverse impacts of both poor growth and overweight/obesity in childhood, and the rapid societal changes in China.

**Added value of this study**

This study adds new information by estimating changes in socio-economic disparities in growth trajectories for height and BMI across recent decades using longitudinal growth data of Chinese children and adolescents from 1991 to 2015. We included a breadth of SEP indicators at both community-level (urbanization) and family-level (household income, parental education, and occupational class). Our analysis shows that children with high SEP had taller stature and greater BMI on average. Large socio-economic disparities in height trajectories (3–5cm) have persisted in the last 25 years, whereas disparities in BMI have widened from ∼<0.5kg/m^2^ to ∼1kg/m^2^ during the same period. Consistent trends were also evident for socio-economic disparities in the upper (overweight/obesity) and lower (stunting/thinness) ends of both height and BMI distributions.

**Implications of all available evidence**

Despite rapid economic growth, large socio-economic disparities in height growth have remained while widening in BMI among Chinese children. These patterns are consistent with findings in many LMICs. These findings demonstrate that the double burden of malnutrition remains stratified by SEP in Chinese youth. In LMICs such as China, effective policy is needed to improve the height growth of children with low-SEP, while also reducing the risk of overweight/obesity, especially of children with high-SEP. For example, efforts are needed to improve nutritional status of rural or low SEP children, as well as the awareness of healthy diet and physical activity for all, including urban or high-SEP children.

Alt-text: Unlabelled box

## Introduction

Growth and development during childhood and adolescence are associated with health and wellbeing across the life-course.^1^ Both short stature^2^ and increased body mass index (BMI)^3^ have important influences on morbidity and mortality in adulthood.^4^ In low-/middle-income countries (LMICs), stunting and underweight (indicators of malnutrition) in children are associated with excess disability^5^ and death,^6^ whereas in some rapid developing societies, increases in childhood overweight and obesity have also become a main driver of later-life cardiovascular risk and mortality.^7^ These outcomes, easily assessed in anthropometric surveys, are therefore key targets of public health policies.

Several recent reviews showed that in high-income countries (HICs), children from families with higher socio-economic position (SEP) had taller stature and lower BMI on average than their poorer counterparts,^8^^,^^9^ whereas in LMICs, children from high SEP or urban sites were taller, but had greater mean BMI.^7^^,^^10^^,^^11^ The contrasting directions of the SEP-BMI association between HICs and LMICs may be due to differential SEP patterns of risk factors. In HICs, for example, individuals with low SEP tend to have higher intake of energy, saturated fat and lower intake of fiber, whereas in many LMICs the opposite trend was observed.^12^ In many HICs, the socio-economic difference in height has narrowed in recent decades,^9^^,^^13^ but disparities in childhood BMI (greater mean BMI in lower SEP groups) have widened.^8^^,^^14^ In LMICs, however, secular trends in socio-economic differences in childhood growth have been inconsistent.^15^ A systematic analysis of data from 141 LMICs at different stages of economic development showed that urban children under 5y were taller and heavier on average than their rural counterparts. The urban-rural difference in height-for-age narrowed in southern/tropical Latin America and South Asia but changed little elsewhere. The difference in weight-for-age also narrowed in southern/tropical Latin America but widened in east/southeast Asia and elsewhere between 1985 and 2011.^16^

In China, there have been consistent trends in recent decades of increasing mean height and decreasing stunting,^1^^,^^17^ but increasing overweight/obesity among children and adolescents.^18^^,^^19^ While China transitioned from a low- to an upper-middle-income country, large disparities persisted between regions in living standards.^20^ The average GDP per capita in coastal (eastern) regions was nearly double of that in inland (western/central) regions by 2020.^21^ We hypothesize that socio-economic differences in child growth have changed over time. As the limited evidence available is inconclusive, it remains unclear whether socio-disparities in growth trajectories have widened, narrowed, or reversed. The Chinese national (cross-sectional) survey on students’ constitution and health (CNSSCH)^22^ reported that urban setting was associated with lower prevalence of stunting and thinness, but higher prevalence of overweight/obesity. Such urban-rural disparities appeared to narrow during 1995–2014,^22^ but in contrast studies using the first eight waves of the China Health and Nutrition Survey (1989–2009) found widening income disparities in childhood overweight/obesity^23^ and height for age z-score.^24^ These studies did not consider the age trajectories for height and BMI, thus disparities in growth at different ages were not fully explored.

Using the longitudinal data of the China Health and Nutrition Survey, including the recent waves in 2011 and 2015, we investigated whether (1) age trajectories for height and BMI differed by community-/household-level SEP indicators among Chinese children and adolescents and (2) socio-economic disparities in growth trajectories changed over time. We also examined whether disparities in the upper (overweight/obesity) and lower (stunting and thinness) ends of height and BMI distributions changed across recent generations.

## Methods

### Study population

The China Health and Nutrition Survey (CHNS) is a longitudinal household survey with ten waves from 1989 to 2015.^25^ Nine provinces (Guangxi, Guizhou, Henan, Hubei, Hunan, Jiangsu, Shandong, Heilongjiang, and Liaoning) were included in all waves. A multistage random cluster sample design was adopted to ensure adequate representation. Within each province, the capital city, a low-income city and four (one high-, two middle-, and one low-income) counties were selected. Two urban and two suburban communities (from each city) or one township and three rural villages (from each county) were selected. Twenty households were selected from each community, town or village. At each wave, community-/household-/individual-level surveys were conducted by trained field workers. Since 1991, all members of selected households have been surveyed (1989-survey included only pre-school children and adults 20–45 years).

Our analyses included 5095 children born between 1981 and 2000, who were 7–18 years with physical measures from 1991 to 2015. We derived four 5-year birth cohorts (born in 1981–85, 1986–90, 1991–95 and 1996–2000) (**Supplemental Table S1**).

All participants provided written informed consent. The University of North Carolina and the Chinese Centre for Disease Control and Prevention reviewed and approved the data collection procedures.^25^

### Measures of body sizes

At each wave, height (to nearest 0.1cm) was measured twice and weight (0.1kg) was measured once in lightweight clothes without shoes using a beam scale and a portable stadiometer respectively.^26^ The average of two height measures was recorded and BMI (kg/m^2^) was calculated. We derived height-for-age (HAZ) and BMI-for-age (BAZ) z-scores using sex-/age-specific WHO 2007 growth reference^27^ to define stunting (HAZ<-2SD), thinness (BAZ<-2SD) and overweight/obesity (BAZ>1SD).

### Measures of socioeconomic position

At each wave, information indicating the level of urbanization of each community (e.g. on infrastructure, services, demographic/economic environment) was obtained from community surveys and classified into 12 components (0–10 points each) (**Supplementary Table S2**). An *urbanization index* was derived as the sum of these components (0–120), with a higher score indicating a higher level of urbanization.

Household income per capita at each wave was calculated as the sum of self-reported annual income of all adult family members divided by household size, and inflated to 2015 Chinese currency (Yuan) by adjusting for the consumer price index ratio.

We used urbanization index and household income per capita at 7 year as indicators of childhood SEP. As not all children were surveyed at 7 year, we estimated individual SEP at 7 year using two-level models:

$$SEP_{ij}=\beta_{0j}+\beta_{1j}\left(t_{ij}-1991\right)+\varepsilon_{ij}$$

where SEP measurements (level 1, *i*) were clustered within level 2 (*j*) units (communities for urbanization index or households for income), and *t_ij_* (=*birth* *year* + 7) represented survey year. We defined high and low SEP groups as ‘≥’ and ‘<’ median urbanization index or median household income per capita estimated at 7 year.^28^

Parental educational attainment and occupation were obtained by linking information reported by adults to children in the same household based on reported relationships. We used parental education and occupational class when children aged 7 year (or the closest age if unavailable) as they were relatively stable in adulthood (95% remained stable). Parental education (highest attainment of parents) was classified into high (≥high school) and low (<high school) education groups. Parental occupational class (paternal occupation, and if missing, proxied by maternal occupation, 2.6%) was classified into high (classes I–IV) and low (class V) SEP groups^29^ (**Supplementary Table S3**).

### Statistical analysis

We first applied two-level linear models (**Supplementary Table S4: model 1**) to height and BMI z-scores (HAZ, BAZ), accounting for the clustering of measurements (level-1) within individuals (level-2). For each SEP indicator, we estimated mean HAZ and BAZ for high (i.e. community urbanization index ≥median, household income per capita ≥median, parental education ≥high school, or occupational classes I-IV) and low SEP groups, and their differences in each cohort.

Second, to assess whether socio-economic differences in age trajectories for height and BMI have changed across cohorts, we explored fractional polynomial functions for age to capture the non-linear growth curves.^30^ Based on Akaike information criterion, Bayesian information criterion, and likelihood test, the best-fitting fractional polynomials for height included age, age^2^, age^3^ for boys; and age^2^, age^2^·ln (age), age^3^ for girls. For BMI, they included age^2^ and age^3^ for both sexes. We adopted the random intercept and coefficients for age terms in models for height and BMI, and included the interactions (age terms × cohort) to allow flexible growth curves across cohorts (**Table S4: models 2–4**). These models allow individuals with different number and timing of measurements, or with incomplete data (i.e. children with a single measure). We tested the interactions (age terms × SEP, cohort × SEP) to assess whether socio-economic disparities in growth differed across ages and cohorts. The higher order terms with *P* >0.05 were removed to achieve the most parsimonious model. For illustration, we depicted mean (95% CIs) trajectories for height and BMI for the earliest (1981–85) and latest (1996–2000) cohorts by SEP groups. To demonstrate the cohort trend in socio-economic disparities, we also estimated the differences in mean (95% CIs) height and BMI between high and low SEP groups at 7, 13 and 17 years for each cohort (**Tables S6.1 and S6.2**).

To assess socio-economic disparities in the upper-/lower-end of height and BMI distributions over time, we applied two-level logistic regressions (adjusting for sex and age) to stunting, thinness, and overweight/obesity (as 1; and non-stunting, non-thinness, non-overweight/obesity as 0, respectively) (**Table S4: model5**). We estimated their odds ratios (ORs, 95% CIs) between high and low SEP groups for each cohort. We also tested the interaction (cohort × SEP) to assess whether socio-economic disparities in stunting, thinness and overweight/obesity changed across cohorts. For illustration, we estimated the prevalence of stunting, thinness and overweight/obesity for boys aged 10 year by SEP group for each cohort.

### Sensitivity analysis

We repeated main analyses (1) using four-level models to assess the impact of clustering within community (level-4) and household (level-3) on socio-economic disparities in growth and (2) including 3,736 children with repeated growth measures to assess the impact of sample attrition.

MLwiN 3.04 was used for multilevel logistic modelling. R 3.6.1 was used for all other analyses (packages mfp, lme4, quantreg and ggplot2 for fractional polynomial, multi-level modelling, and plots).

### Role of the funding source

The funders of the study had no role in study design, in the collection, analysis and interpretation of data, in the writing of the report, and in the decision to submit the paper for publication.

## Results

In the study sample, 3736 (73%) children had ≥two, and 1985 (40%) had ≥three repeated measurements (maximum five measurements per child with an average of 2.24). Mean height and BMI z-scores (HAZ and BAZ) have increased across cohorts. Between the earliest and latest cohorts, the rate of stunting decreased (from 21.5% to 10.1%), while overweight/obesity increased (6.8%–18.2%). The rate of thinness changed little. The distributions of childhood SEP measures have changed. The median of urbanization index increased from 43.5 to 56.2, the median of household income per capita increased rapidly from 2362 to 5622 Yuan. More parents had a non-manual occupation (34.7%–40.2%). There was a decline in parents with educational levels of high school or above (34.3% to 28.4%). However, mean schooling year increased during the period (from 8.6 to 9.3 years) (Table 1) due the increase in parents who finished middle-school (39%–54.8%).

**Table 1:** Summary statistics for measures of physical growth and SEP for each cohort.

Cohort	1981–85	1986–90	1991–95	1996–2000
**Growth measures**				
N (measurements)†	3767	3816	2094	1883
Height (HAZ) - mean (SD)	-1.12 (1.11)	-0.85 (1.14)	-0.61 (1.13)	-0.45 (1.22)
BMI (BAZ) - mean (SD)	-0.55 (1.04)	-0.44 (1.14)	-0.39 (1.11)	-0.25 (1.32)
Stunting (HAZ<-2SD) (%)	21.5%	15.8%	11.4%	10.1%
Thinness (BAZ<-2SD) (%)	7.2%	7.5%	6.4%	8.0%
Overweight (BAZ>1SD) (%)	6.8%	9.9%	11.4%	18.2%
**SEP indicators**§				
N (individuals)ǂ	1560	1767	956	835
Urbanization index				
Median	43.5	48.0	51.9	56.2
Range	19.3-80.1	23.4-83.6	27.9-89.0	33.1-94.6
Household income per capita*				
Median	2362	3231	4225	5622
Range	947–5416	913–12084	758–15651	561–23388
Parental education				
<High school (%)	65.7%	64.4%	69.2%	71.6%
Mean schooling year-Mean (SD)	8.6 (3.2)	9.2 (2.8)	9.3 (2.7)	9.3 (2.7)
Parental occupation				
Class V (%)	65.3%	64.6%	63.6%	59.8%

Abbreviations: SEP – socio-economic position.

§ Missing data (% of 5095 individuals): <0.3% for income, <3.0% for parental education, and 4.1% for occupation; <2% for BMI; no missing for other measures

† Growth: measurements as a unit. HAZ and BAZ were height-for-age and BMI-for-age z-scores using sex- and age-specific WHO 2007 growth reference.

ǂ SEP indicators: individual as a unit.

⁎ In Chinese currency (Yuan).

**Figure 1:**
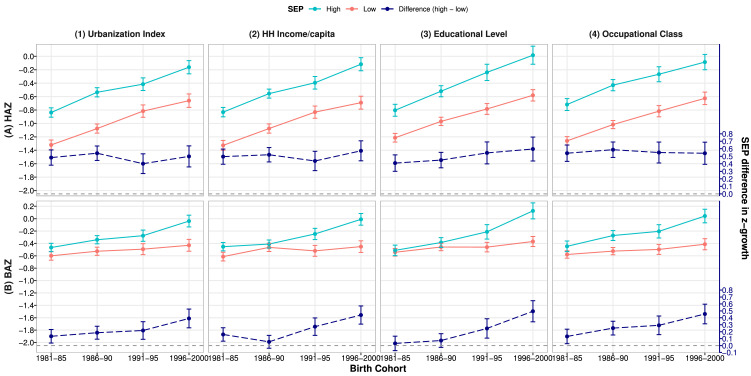
Mean (95%CI) height and BMI z-scores (HAZ and BAZ)^§^ by SEP groups^†^ and SEP differences (high – low) in z-scores across cohorts^‡^ Note: 1981–85: earliest cohort, 1996–2000: latest cohort. Abbreviations: HH: household; SEP: socio-economic position; CI: confidence interval. ^§^HAZ and BAZ were height-for-age and BMI-for-age z-scores using sex- and age-specific WHO 2007 growth reference. ^†^High/low SEP groups: urbanization Index and HH income per capita: ≥/< cohort-specific median. Educational level: ≥ High/≤ Middle school. Occupational class: class I-IV /V. ^‡^All values were estimated from 2-level linear models for height and BMI z-scores (level-1: measurement; level-2: individual).

Figure 1 shows the mean HAZ and BAZ by SEP groups and differences in HAZ and BAZ between high and low SEP groups (corresponding values in **Supplementary Table S5**). In all cohorts, children were on average taller in high than low SEP groups, by a magnitude of 0.40–0.60 HAZ (*P*<0.05) depending on the SEP indicator (upper panel). The trends of increasing height were similar for high and low SEP groups, thus large socio-economic differences in mean HAZ persisted across cohorts (dash line corresponding to right y-axis). For example, the difference was 0.48(95% CI: 0.38,0.58) in the earliest and 0.50(0.36,0.64) in latest cohort between high and low urbanization index groups (upper panel (1)), and 0.50(0.39,0.60) and 0.57(0.44,0.71) respectively between high and low household income groups (upper panel (2)). Children in high SEP groups also had greater mean BMI z-score than their low-SEP counterparts for most SEP indicators (lower panel). The trends of increasing BAZ were greater in high than in low SEP groups, thus the socio-economic differences in mean BAZ widened (dash line). For example, the difference increased from 0.13(0.04,0.23) in the earliest to 0.39(0.26,0.53) in the latest cohort by urbanization index group (lower panel (1)) and from 0.16(0.06,0.26) to 0.44(0.31,0.57) by household income group (lower panel (2)).

### Socio-economic disparities in height trajectories

**Figure 2:**
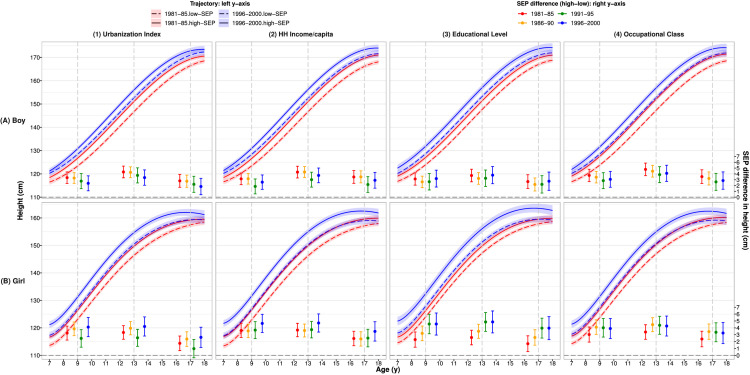
Mean height (95%CI) trajectories by SEP groups* for the earliest & latest cohorts (left y-axis) and differences in mean height between high and low SEP groups (right y-axis) across cohorts for (A) boys and (B) girls^†^ Note: 1981–85: earliest cohort, 1996–2000: latest cohort. Abbreviations: HH: household; SEP: socio-economic position; CI: confidence interval. *High/low SEP group: urbanization Index and HH income per capita: ≥/< cohort-specific median. Educational level: ≥ High/<high school. Occupational class: class I–IV /V. ^†^All values were estimated from 2-level fractional polynomial models (level-1: measurement; level-2: individual).

Figure 2 shows an upward shift of mean height trajectories from the earliest to the latest cohort. In each cohort, height trajectories of children in high SEP groups were above those of low SEP groups throughout childhood to late adolescence in both sexes (blue/red bars corresponding values in **Supplementary Table S7**). Socio-economic disparities in linear growth were similar across cohorts and SEP indicators (blue/red bars corresponding to right y-axis). For example, at 10y, differences in mean height between high and low urbanization index groups and household income groups ranged from 2.9cm(1.6,4.1) to 3.8cm(2.9,4.8) for boys and from 3.4cm(2.4,4.3) to 4.7cm(3.4,6.0) for girls in all cohorts. In both the earliest and latest cohorts, height disparities varied by age, were greatest during adolescence and narrowed slightly thereafter, with a smaller difference in late adolescence. For example, for boys, the difference in mean height (by urbanization index or household income groups) was 4.3cm at 13y, reduced to 2.8∼3.5cm at 17y in the earliest cohort (red bars, upper panel (1)-(2)), and in the latest cohort reduced from 3.4∼3.7cm to 1.8∼2.9cm, respectively (blue bars, upper panel (1)-(2)). Socio-economic differences were similar for girls, 3.3∼3.7cm at 13y, reduced to 1.7∼2.5cm at 17y in the earliest cohort, and in the latest cohort reduced from 4.2∼4.7cm to 2.6∼3.5cm (red/blue bars, lower panel (1)-(2), **Supplementary Table S6.1**).

### Socio-economic disparities in BMI trajectories

**Figure 3:**
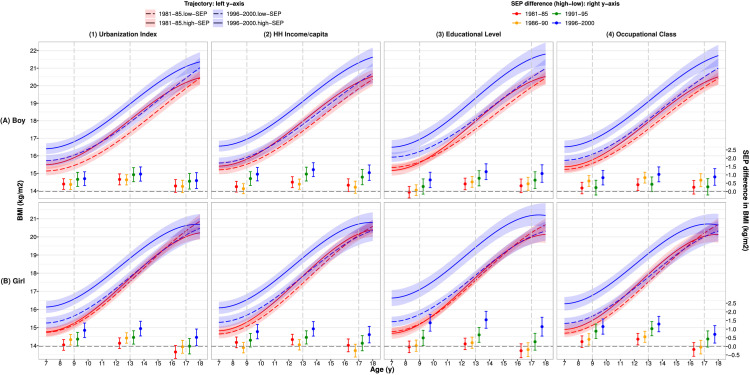
Mean BMI (95%CI) trajectories by SEP groups* for the earliest & latest cohorts (left y-axis) and differences in mean BMI between high and low SEP groups (right y-axis) across cohorts for (A) boys and (B) girls^†^ Note: 1981–85: earliest cohort, 1996–2000: latest cohort. Abbreviations: HH: household; SEP: socio-economic position; CI: confidence interval. *High/low SEP group: urbanization Index and HH income per capita: ≥/< cohort-specific median. Educational level: ≥ High/<high school. Occupational class: class I-IV /V. ^†^All values were estimated from 2-level fractional polynomial models (level-1: measurement; level-2: individual).

Figure 3 shows mean BMI trajectories in the latest cohort were also above those in the earliest cohort and socio-economic disparities in BMI growth were much greater in the latest than earliest cohort (blue vs red bars, corresponding values in **Supplementary Table S7**). For example, at 10 year, socio-economic differences by household income group increased from 0.4kg/m^2^(0,0.7) in the earliest cohort to 1.1kg/m^2^(0.7,1.5) in the latest cohort in boys (upper panel (2)), and in girls, from 0.3kg/m^2^(0,0.6) to 0.9kg/m^2^(0.5,1.3) respective (lower panel (2)). Disparities in BMI were greatest during adolescence and narrowed slightly thereafter. For example, in boys, socio-economic difference in mean BMI (by urbanization index and household income groups) was 0.6∼0.7kg/m^2^ at 13y, decreased to 0.3∼0.4kg/m^2^ at 17y in the earliest cohort (red bars, upper panel (1)-(2)), and in the latest cohort, decreased from 1.0∼1.3kg/m^2^ to 0.7-1.1kg/m^2^ respectively (blue bars, upper panel (1)-(2)). Findings were similar for girls: the difference reduced from 0.2∼0.4kg/m^2^ to -0.3∼0.1kg/m^2^ in the earliest cohort, and from 1.0kg/m^2^ to 0.5∼0.7kg/m^2^ in the latest cohort (red/blue bars, lower panel (1)-(2)**, Supplementary Table S6.2**).

### Socio-economic disparities in stunting, thinness, and overweight/obesity

**Figure 4:**
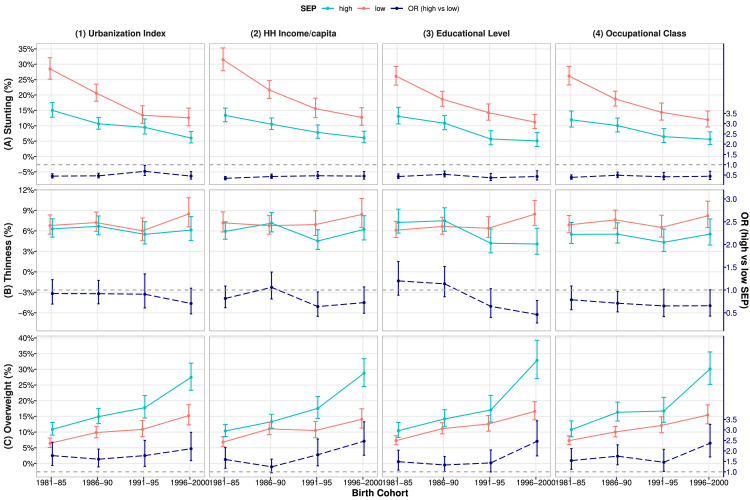
Prevalence (95%CI) of stunting, thinness and overweight/obesity § by SEP groups (solid lines) * and their ORs (high vs low SEP, dash lines) across cohorts^†^ Note: 1981–85: earliest cohort, 1996–2000: latest cohort. Abbreviations: HH: household; SEP: socio-economic position; OR: odds ratio; CI: confidence interval. ^§^Stunting, thinness and overweight were defined by WHO 2007 reference. * High/low SEP groups: urbanization Index and HH income per capita: ≥/< cohort-specific median. Educational level: ≥ High/≤ Middle school. Occupational class: class I-IV /V. ^†^All values estimated for **boys aged 10 year** from 2-level (level-1: measurement; level-2: individual) logistic models with adjustment for age and sex.

Figure 4 shows the prevalence of stunting, thinness and overweight/obesity and their ORs by SEP groups (corresponding values in **Supplementary Table S8**). Higher SEP was associated with reduced risk of stunting: OR=0.49(0.42,0.56) for children from high (vs low) urbanization index group and 0.41(0.35,0.47) for high (vs low) household income group (upper panel (1)-(2)). The risk of stunting decreased substantially across cohorts (*P*<0.05) in both low (e.g. from 28.5–31.5% in the earliest to 12.7% in the latest cohort for 10-year-old boys) and high SEP groups (13.4–15% to 6.0%, upper panel (1)–(4)). Thus, socio-economic disparities in stunting persisted across cohorts (dash lines, upper panel).

There was no association between childhood SEP and thinness (middle panel), but higher SEP was associated with increased risk of overweight/obesity: OR=1.77(1.52,2.06) and 1.64(1.41,1.90) for high (vs low) SEP defined by urbanization index and household income, respectively (lower panel (1)-(2)). The prevalence of overweight/obesity increased substantially from the earliest to the latest cohort (*P*<0.05) in both low (i.e. for 10-year-old boys, from 6.4∼6.8% to 14.1∼15.2%) and high SEP groups (from 10.4∼10.9% to 27.4∼28.8%, lower panel (1)–(4)). The increasing trends were greater in high than those in low SEP group, thus disparities in overweight/obesity have widened over time (*P*<0.05, dash lines, lower panel): ORs increased from 1.59∼1.77 in the earliest to 2.10∼2.46 in the latest cohort (**Supplementary Table S8**).

### Sensitivity analysis

Analyses using four-level models showed smaller socio-economic differences in mean height and BMI, as part of differences were explained by communities (level-4). However, trends in socio-economic disparities in height and BMI trajectories across cohorts were similar to those estimated using two-level models. Thus, we presented findings from two-level models. Additional analysis including children with ≥two measurements showed similar results as main findings (**data not shown**).

## Discussion

Using longitudinal anthropometric measurements of Chinese children/adolescents from 1991 to 2015, we found that higher SEP was associated with greater mean height and BMI trajectories. Consequently, large disparities in height trajectories (equivalent to ∼3–5cm for 10-year-olds) persisted across cohorts and SEP indicators. Moreover, socio-economic differences in BMI widened, from ≤0.5kg/m^2^ in the earliest to ∼1kg/m^2^ in the latest cohort. Consistent with mean height and BMI, socio-economic differences in stunting persisted while differences in overweight/obesity widened across cohorts.

### SEP and growth trajectories for height and BMI

The associations of high SEP with taller stature and a lower risk of stunting found in our study have been reported in both HICs and LMICs.^14^^,^^16^^,^^31^ Urban children were found to be taller than rural children in many LMICs.^16^ Analyzing both household- and community-level SEP markers, our study showed greater mean height trajectories for children in high (vs low) SEP groups, with the height increment between the earliest and latest cohorts greater around adolescence than at other ages. This pattern was also observed in a study using two large cross-sectional surveys for Chinese children/students between 1975 and 2010, suggesting faster growth tempo and earlier maturation in children with higher SEP (defined by gross national income and life expectancy).^32^

In our study, mean BMI trajectories and risk of overweight/obesity were greater for children in high (vs low) SEP groups across SEP measures, especially children born more recently. Like patterns for height, socio-economic disparities in BMI increased with age during childhood, narrowed in late-adolescence. The positive SEP-BMI association is consistent with a study using the CNSSCH which found higher obesity prevalence in urban than rural children (7-18y).^33^ Another study using the CHNS waves 1991-2011 showed that children (1-17y) with higher SEP (top quintile of urbanization index) had higher BMI z-scores than those with lower SEP.^34^ This positive association was reported in most LMICs,^16^ but contrasts with the direction observed in HICs.^8^^,^^9^ Obesity risk factors and their associations with SEP in LMICs may differ from those in HICs.^35^ In HICs, children from affluent families are more likely to have healthy diet and regular exercise,^8^ whereas in LMICs, children from higher SEP or urbanized areas have more access to energy-dense food and components of a sedentary lifestyle (e.g. passive commuting, long screen time and reduced sleep).^7^^,^^10^ Furthermore, children from higher SEP are both taller and heavier, indicating that BMI and height are positively correlated.^36^ Our findings of high SEP being associated with overweight/obesity rather than lower BMI (as in HICs) indicate a right-skewed BMI distribution for children with high SEP.

### Changes in socio-economic disparities in growth trajectories over time

Comparing children born in 1981–85 with those born in 1996–2000, we found a large increase in height (∼5cm) and the prevalence of overweight/obesity (6.8%–18.2%), a modest increase in mean BMI (0.5∼1.3kg/m^2^), and a decrease in stunting (21.5% to 10.1%), but little change in thinness. These changes are consistent with those reported in two national surveys, except for the trend for thinness in the CNSSCH, which decreased between 1985 and 2014.^17^^,^^32^ Increases in community urbanization (e.g. improvement in infrastructure and health service) and household income have contributed to the trends in growth in Chinese children.^37^ However, these improvements may have co-occurred with increasing unhealthy lifestyles (consumption of energy-dense food, less fruits/vegetables^38^ and sedentary behavior)^39^ which are risk factors for obesity.^40^

Given the rapid economic development in China, we would expect disparities in growth in Chinese children to have started to follow the patterns in many HICs,^15^ i.e. narrowing the socio-economic difference in height while also narrowing (or reversing) the gaps in BMI (i.e. higher BMI in low rather than high SEP groups). Previous studies of trends in socio-economic disparities in height of Chinese children were inconclusive, reporting either persistent^41^ or narrowing^22^ urban-rural differences in height. Studies using CHNS waves 1989–2009 showed widening socio-economic disparities in childhood height.^24^ The discrepancies in these findings may be due to different SEP indicators used, such as household net income quintiles, provincial level socio-economic characteristics,^24^ and community context (top quintile of urbanization index).^34^ Our study helps resolve this inconsistency, by showing persistent disparities in height trajectories across a range of community-/family-level SEP indicators. This finding is consistent with those found in some LMICs (east and southeast Asia),^16^ but contrasts with recent data from HICs which showed diminishing height inequalities.^13^^,^^14^

Our finding of widening socio-economic disparities in BMI trajectories in recent generations is consistent with studies from both LMICs^16^ and the CHNS during 1991–2011.^23^^,^^34^ However, studies using CNSSCH, a large successive national survey from 29 provinces in China, reported narrowing/diminishing trends in urban-rural disparities in obesity^22^^,^^33^ due to greater increasing trends in rural children. The CNSSCH used a cross-sectional design and aggregated regional-level SEP measures (e.g. GDP and administrative urban-rural classification),^22^ while our study used longitudinal data and also family-level SEP measures (household income, parental education, occupational class). Findings from previous reviews on the SEP-obesity association^42^ indicated that the positive relation reversed when a country developed into a higher economic category. Similar to findings from some LMICs,^16^^,^^43^ however, we found that socio-economic differences in BMI did not narrow, or reverse to follow the pattern of HICs.^8^^,^^9^^,^^14^ Meanwhile, our additional analysis on the BMI of parents showed that disparities in BMI (high BMI for high SEP) had narrowed in fathers and reversed in mothers in the latest cohort (data not shown). As found in a recent review,^44^ in some middle-income countries^43^ the prevalence of obesity for women had shifted from high- to low-SEP groups, but not for men and children. Shifts in the SEP-BMI association may occur more slowly for men than for women, and may not be evident in children until the country has experienced a sustained higher economic level.^44^

### Implications

Our findings of the persistent associations of short stature with lower SEP, but higher BMI with higher SEP in Chinese children and adolescents in recent 25 years have important implications for China and LMICs experiencing rapid economic development with similar patterns of disparities in child growth. Effective policy is needed to improve nutritional status and thus, height growth of rural or low SEP children. Public health efforts are also required to improve the awareness of healthy diet and physical activities to reduce the risk of overweight/obesity among all children, including urban or high-SEP children.

### Strengthens and limitations

Our study has several strengthens: First, we applied random-effects modelling to repeated growth measurements to assess socio-economic disparities in growth trajectories using a longitudinal survey spanning over 24 years. This allowed us to explore the impact of within-community, within-household, and within-individual clustering on our findings. Second, we went beyond previous studies by considering a breadth of family-/community-level SEP indicators that reflect different, but relevant socio-economic characteristics.

However, limitations existed: First, loss to follow-up occurred over time and were 12–20% across waves. We applied random-effect models that allowed children with different numbers and timing of measurements. Sensitivity analysis of children with ≥two measurements showed similar findings. Second, there was a relatively small number of older adolescents in the latest cohort, which might explain their wider 95% CIs for estimated growth trajectories. This limitation can be addressed using future CHNS waves. Last, Asians tend to have a higher percentage of body fat and bear greater metabolic burden than White European populations with the same BMI.^45^ The use of BMI cannot distinguish fat and fat-free mass and these two tissues may have different health implications in later life. Future studies with fat measures are needed to estimate the socioeconomic disparities in fat mass.

## Conclusions

Despite recent economic growth in China, socio-economic disparities in physical growth exist among Chinese youths and the patterns reflect those in LMICs. Large disparities in height (i.e. short stature for lower SEP children) have persisted, whereas disparities in BMI (high BMI for higher SEP children) widened in recent decades. Our data highlight that, unlike in HICs, the double burden of malnutrition in China remains stratified by SEP. Both low and high SEP groups would benefit from public health initiatives tailored to the children's circumstances in China and also other rapidly developing LMICs.

## Data sharing statement

The data used in this study can be obtained from cpc.unc.edu/projects/china.

## Contributors

LL and MG conceived the study and designed the analysis. JW and WJ made suggestions on the study design. MG analyzed the data and wrote the first draft of the manuscript. All authors edited and revised the paper, contributed to the interpretation of data, and approved the final version.

## Funding

MG is supported by UCL Overseas Research Scholarship and China Scholarship Council for her PhD study. WJ is supported by a UK Medical Research Council (MRC) New Investigator Research Grant (MR/P023347/1) and acknowledges support from the National Institute for Health Research (NIHR) Leicester Biomedical Research Centre, which is a partnership between University Hospitals of Leicester NHS Trust, Loughborough University, and the University of Leicester.

## Declaration of interests

We declare no competing interests.

## Appendix Supplementary materials

Image, application 1
