# Supplementary material for: Socio-economic disparities in child-to-adolescent growth trajectories in China: Findings from the China Health and Nutrition Survey 1991–2015
Source: Lancet Reg Health West Pac. 2022 Feb 26;21:100399. doi: 10.1016/j.lanwpc.2022.100399 (PMC9079352; doi:10.1016/j.lanwpc.2022.100399)
Supplement: Supplementary file 1 [file mmc1.docx]

# Supplementary material

**Table S1.** Number of children† in each survey year for each 5-year cohort

| **Birth year** | **1991** | **1993** | **1997** | **2000** | **2004** | **2006** | **2009** | **2011** | **2015** | **Total**ǂ | **Number of measurements per child** | | | | **Mean (SD)** | **Median**  **(IQR)** | **Total** |
| --- | --- | --- | --- | --- | --- | --- | --- | --- | --- | --- | --- | --- | --- | --- | --- | --- | --- |
|  |  |  |  |  |  |  |  |  |  |  | One | Two | Three | Four/Five |  |  |  |
| **1981-85** | **880** | **1115** | **1018** | **672** |  |  |  |  |  | **3697** | 319 (20.5%) | 523 (33.7%) | 512 (33%) | 199 (12.8%) | 2.38 (0.95) | 2  (2-3) | **1553** |
| Age (y) | 7-10 | 8-12 | 12-16 | 15-18 |  |  |  |  |  |  |  |  |  |  |  |  |  |
| **1986-90** |  | **225** | **1188** | **1337** | **731** | **292** |  |  |  | **3765** | 504 (28.6%) | 649 (36.9%) | 469 (26.6%) | 139 (7.9%) | 2.14 (0.92) | 2  (1-3) | **1761** |
| Age (y) |  | 7 | 7-11 | 10-14 | 14-18 | 16-18 |  |  |  |  |  |  |  |  |  |  |  |
| **1991-95** |  |  |  | **408** | **627** | **522** | **359** | **152** |  | **2064** | 300 (31.6%) | 302 (31.8%) | 243 (25.6%) | 105 (11.1%) | 2.17 (1.02) | 2  (1-3) | **950** |
| Age (y) |  |  |  | 7-9 | 9-13 | 11-15 | 14-18 | 16-18 |  |  |  |  |  |  |  |  |  |
| **1996-2000** |  |  |  |  | **209** | **435** | **543** | **458** | **218** | **1863** | 236 (28.4%) | 277 (33.3%) | 215 (25.9%) | 103 (12.4%) | 2.24 (1.04) | 2  (1-3) | **831** |
| Age (y) |  |  |  |  | 7-8 | 7-10 | 9-13 | 11-15 | 15-18 |  |  |  |  |  |  |  |  |
| **Totalǂ** | **880** | **1340** | **2206** | **2417** | **1567** | **1249** | **902** | **610** | **218** | **11389** | 1359 (26.7%) | 1751 (34.4%) | 1439 (28.2%) | 546 (10.7%) | 2.24 (0.97) | 2  (1-3) | **5095** |

† Same child could appear in multiple surveys

ǂ Number of measurements

**Table S2.** Twelve components (scale 0-10) of urbanicity^[1]^ of communities in China Health and Nutrition Survey

| **Component** | **Details** |
| --- | --- |
| **Population density** | -Total population of community/area, from official records |
| **Economic activity** | -Typical daily wage for ordinary male workers, reported by community officials  -% population engaged in non-agricultural work |
| **Traditional markets** | -Distance to market in 3 categories (within boundaries of the community, within the city but not in this community or not within the city/ village/town)  -No. days of operation for 8 types of market (including food and fuel markets) |
| **Modern markets** | -No. supermarkets, cafés, Internet cafés, indoor restaurants, outdoor fixed/mobile eateries, bakeries, ice cream parlours, fast food restaurants, fruit/vegetable stands, bars within community boundaries |
| **Transportation infrastructure** | -Most common type of road  -Distance to bus/train stop, categorized as within≤1 km or ≥1 km from the community |
| **Sanitation** | -% households with treated water  -% households without excreta present outside home |
| **Communications** | -Availability (within community boundaries) of cinema, newspaper, postal or telephone service  -% households with a computer  -% households with a television  -% households with a cell phone |
| **Housing** | -Average No. days/week that electricity is available to the community  -% community with indoor tap water  -% flush toilets, % cooks with gas |
| **Education** | -Average education level among adults >21 years old |
| **Diversity** | -Variation in community education and income levels |
| **Health infrastructure** | -Number & type of health facilities in or nearby (≤12 km) the community  -Number pharmacies in the community |
| **Social services** | -Provision of preschool for children <3y and availability of (offered in community) commercial medical insurance, free medical insurance and/or insurance for women and children |

^[1]^ Jones-Smith JC, Popkin BM. Understanding community context and adult health changes in China: development of an urbanicity scale. Social Science and Medicine. 2010;71(8):1436-46.

**Table S3.** Social class based on self-reported primary occupation

| **Social class** | **Primary occupation** |
| --- | --- |
| Class I | 1. Senior professional/technical worker |
| Class II | 1. Junior professional/technical worker |
|  | 2. Administrator/executive/manager |
|  | 3. Army officer, police officer |
| Class III-M (skilled manual) | 1. Skilled worker |
| Class III-NM (skilled non-manual) | 2. Office staff |
|  | 3. Soldier, policeman |
|  | 4. Athlete, actor, musician |
| Class IV (semi-skilled) | 1. Driver |
|  | 2. Service worker |
| Class V (unskilled) | 1. Farmer, fisherman, hunter |
|  | 2. Non-skilled worker |

**Table S4.** Random-effects regression models used in this study

| **Random-effects models for z-score of height/BMI (1)** |
| --- |
| ${z-score}_{ij}=\alpha_{0j}+\delta_{0}{SEP}_{j}+\sum_{k=2}^{4} \gamma_{ko}C_{k}+ \sum_{k=2}^{4} \theta_{ko}C_{k}\cdot{SEP}_{j}+\varepsilon_{ij}$ (1) |
| **Random-effects fractional polynomial models for height trajectories for boys (2) and girls (3)** |
| ${HT}_{ij}=\alpha_{0j}+\beta_{1j}{age}_{ij}+\beta_{2}{age}_{ij}^{2}+\beta_{3}{age}_{ij}^{3}+\sum_{k=2}^{4} \gamma_{ko}C_{k}+\sum_{k=2}^{4} \gamma_{k1}C_{k}\cdot{age}_{ij}+\sum_{k=2}^{4} \gamma_{k2}C_{k}\cdot{age}_{ij}^{2}+\delta_{0}{SEP}_{j}+\delta_{1}{SEP}_{j}\cdot{age}_{ij}+{\delta_{2}SEP}_{j}\cdot{age}_{ij}^{2}+\sum_{k=2}^{4} \theta_{ko}C_{k}\cdot{SEP}_{j}+\varepsilon_{ij}$ (2) |
| ${HT}_{ij}=\alpha_{0j}+\beta_{1j}{age}_{ij}^{2}+\beta_{1}{age}_{ij}^{2}\cdot ln\left( age \right)+\beta_{3}{age}_{ij}^{3}+\sum_{k=2}^{4} \gamma_{ko}C_{k}+\sum_{k=2}^{4} \gamma_{k1}C_{k}\cdot{age}_{ij}^{2}+\sum_{k=2}^{4} \gamma_{k2}C_{k}\cdot{age}_{ij}^{2}\cdot ln\left( age \right)+\delta_{0}{SEP}_{j}+\delta_{1}{SEP}_{j}\cdot{age}_{ij}^{2}+{\delta_{2}SEP}_{j}\cdot{age}_{ij}^{2}\cdot ln\left( age \right)+\sum_{k=2}^{4} \theta_{ko}C_{k}\cdot{SEP}_{j}+\varepsilon_{ij}$ (3) |
| **Random-effect fractional polynomial models for BMI trajectories for both genders (4)** |
| ${BMI}_{ij}=\alpha_{0j}+\beta_{1j}{age}_{ij}^{2}+\beta_{2}{age}_{ij}^{3}+\sum_{k=2}^{4} \gamma_{ko}C_{k}+\sum_{k=2}^{4} \gamma_{k1}C_{k}\cdot{age}_{ij}^{2}+\delta_{0}{SEP}_{j}+\delta_{1}{SEP}_{j}\cdot{age}_{ij}^{2}+{\delta_{2}SEP}_{j}\cdot{age}_{ij}^{3}+\sum_{k=2}^{4} \theta_{ko}C_{k}\cdot{SEP}_{j}+\varepsilon_{ij}$ (4) |
| **Random-effects logistic regression models for stunting, thinness, overweight and obesity with adjustment for gender and age (5)** |
| ${logit(Y)}_{ij}=\alpha_{0j}+\beta_{1}{age}_{ij}+\beta_{2}{gender}_{j}+\sum_{k=2}^{4} \gamma_{ko}C_{k}+\delta_{0}{SEP}_{j}+\sum_{k=2}^{4} \theta_{ko}C_{k}\cdot{SEP}_{j}+\varepsilon_{ij}$ (5) |

**Table S5.** Estimated mean (95%CI) z-score of height and BMI (HAZ and BAZ) ^§^ by SEP groups^‡^ and their SEP differences (high – low) across cohorts†

|  |  | **z-height** |  |  | **z-BMI** |  |
| --- | --- | --- | --- | --- | --- | --- |
| **cohort** | **High** | **Low** | **High - Low** | **High** | **Low** | **High - Low** |
| **Urbanization Index** | **≥ median** | **< median** |  | **≥ median** | **< median** |  |
| 1981-85 | -0.84 (-0.91, -0.77) | -1.32 (-1.39, -1.25) | 0.48 (0.38, 0.58) | -0.47 (-0.53, -0.4) | -0.60 (-0.67, -0.53) | 0.13 (0.04, 0.23) |
| 1986-90 | -0.54 (-0.6, -0.47) | -1.08 (-1.14, -1.01) | 0.54 (0.44, 0.64) | -0.34 (-0.41, -0.27) | -0.53 (-0.59, -0.46) | 0.19 (0.09, 0.28) |
| 1991-95 | -0.41 (-0.51, -0.32) | -0.82 (-0.91, -0.72) | 0.40 (0.27, 0.53) | -0.28 (-0.37, -0.18) | -0.49 (-0.58, -0.4) | 0.22 (0.09, 0.34) |
| 1996-2000 | -0.16 (-0.26, -0.06) | -0.66 (-0.76, -0.56) | 0.50 (0.36, 0.64) | -0.04 (-0.13, 0.06) | -0.43 (-0.53, -0.33) | 0.39 (0.26, 0.53) |
| **Linear trend*** | 0.22 (0.18, 0.25)* | 0.23 (0.19, 0.27)* | -0.01 (-0.06, 0.04) | 0.13 (0.09, 0.17)* | 0.05 (0.02, 0.09)* | 0.08 (0.02, 0.13)* |
| **Last-first cohort** | 0.68 (0.55, 0.8)* | 0.66 (0.54, 0.78)* | 0.02 (-0.16, 0.19) | 0.43 (0.31, 0.54)* | 0.17 (0.05, 0.29)* | 0.26 (0.09, 0.42)* |
| **HH Income/capita** | **≥ median** | **< median** |  | **≥ median** | **< median** |  |
| 1981-85 | -0.83 (-0.9, -0.76) | -1.33 (-1.4, -1.25) | 0.50 (0.39, 0.60) | -0.45 (-0.52, -0.39) | -0.61 (-0.68, -0.54) | 0.16 (0.06, 0.26) |
| 1986-90 | -0.56 (-0.62, -0.49) | -1.08 (-1.14, -1.01) | 0.52 (0.43, 0.62) | -0.41 (-0.48, -0.34) | -0.46 (-0.53, -0.4) | 0.05 (-0.04, 0.15) |
| 1991-95 | -0.39 (-0.49, -0.3) | -0.83 (-0.92, -0.74) | 0.44 (0.31, 0.57) | -0.25 (-0.34, -0.16) | -0.52 (-0.61, -0.43) | 0.27 (0.15, 0.4) |
| 1996-2000 | -0.12 (-0.21, -0.02) | -0.69 (-0.79, -0.59) | 0.57 (0.44, 0.71) | -0.01 (-0.11, 0.08) | -0.45 (-0.55, -0.36) | 0.44 (0.31, 0.57) |
| **Linear trend*** | 0.23 (0.19, 0.27)* | 0.22 (0.18, 0.26)* | 0.01 (-0.04, 0.06) | 0.14 (0.11, 0.18)* | 0.04 (0.01, 0.08)* | 0.10 (0.05, 0.15)* |
| **Last-first cohort** | 0.71 (0.59, 0.83)* | 0.64 (0.52, 0.76)* | 0.08 (-0.09, 0.24) | 0.44 (0.33, 0.56)* | 0.16 (0.04, 0.28)* | 0.28 (0.12, 0.44)* |
| **Educational Level** | **≥ High school** | **≤ Middle school** |  | **≥ High school** | **≤ Middle school** |  |
| 1981-85 | -0.80 (-0.89, -0.71) | -1.21 (-1.28, -1.15) | 0.41 (0.3, 0.52) | -0.51 (-0.6, -0.43) | -0.54 (-0.6, -0.48) | 0.03 (-0.07, 0.14) |
| 1986-90 | -0.52 (-0.6, -0.44) | -0.97 (-1.03, -0.91) | 0.45 (0.35, 0.55) | -0.39 (-0.46, -0.31) | -0.46 (-0.52, -0.4) | 0.07 (-0.03, 0.17) |
| 1991-95 | -0.24 (-0.36, -0.12) | -0.78 (-0.86, -0.7) | 0.54 (0.4, 0.69) | -0.21 (-0.33, -0.1) | -0.46 (-0.54, -0.38) | 0.25 (0.11, 0.39) |
| 1996-2000 | 0.02 (-0.12, 0.15) | -0.58 (-0.67, -0.5) | 0.60 (0.44, 0.76) | 0.13 (0, 0.25) | -0.37 (-0.45, -0.29) | 0.49 (0.34, 0.65) |
| **Linear trend*** | 0.28 (0.23, 0.32)* | 0.21 (0.18, 0.24)* | 0.07 (0.01, 0.13)* | 0.20 (0.15, 0.25)* | 0.05 (0.02, 0.08)* | 0.15 (0.09, 0.2)* |
| **Last-first cohort** | 0.82 (0.66, 0.98)* | 0.63 (0.53, 0.74)* | 0.19 (-0.01, 0.38) | 0.64 (0.48, 0.79)* | 0.17 (0.07, 0.28)* | 0.46 (0.28, 0.65)* |
| **Occupational Class** | **Classes I-IV** | **Class V** |  | **Classes I-IV** | **Class V** |  |
| 1981-85 | -0.72 (-0.81, -0.63) | -1.26 (-1.32, -1.2) | 0.54 (0.43, 0.65) | -0.45 (-0.53, -0.36) | -0.58 (-0.64, -0.52) | 0.13 (0.03, 0.24) |
| 1986-90 | -0.43 (-0.51, -0.35) | -1.02 (-1.08, -0.96) | 0.59 (0.48, 0.69) | -0.27 (-0.35, -0.19) | -0.52 (-0.58, -0.47) | 0.25 (0.15, 0.35) |
| 1991-95 | -0.27 (-0.38, -0.16) | -0.82 (-0.9, -0.73) | 0.55 (0.41, 0.69) | -0.20 (-0.31, -0.1) | -0.50 (-0.58, -0.42) | 0.29 (0.16, 0.43) |
| 1996-2000 | -0.09 (-0.2, 0.03) | -0.63 (-0.72, -0.53) | 0.54 (0.39, 0.69) | 0.04 (-0.07, 0.15) | -0.41 (-0.5, -0.32) | 0.46 (0.31, 0.6) |
| **Linear trend*** | 0.21 (0.16, 0.25)* | 0.21 (0.18, 0.25)* | 0 (-0.06, 0.05) | 0.15 (0.11, 0.2)* | 0.05 (0.02, 0.08)* | 0.10 (0.05, 0.16)* |
| **Last-first cohort** | 0.63 (0.49, 0.78)* | 0.63 (0.52, 0.75)* | 0 (-0.18, 0.18) | 0.49 (0.35, 0.63)* | 0.17 (0.06, 0.27)* | 0.32 (0.15, 0.5)* |

Abbreviations: HH: household; SEP: socio-economic position; CI: confidence interval.

§ HAZ and BAZ were height-for-age and BMI-for-age z-scores using sex- and age-specific WHO 2007 growth reference.

† All values were estimated from 2-level models (level-1: measurement; level-2: individual).

‡ High/low SEP groups: urbanization Index and HH income per capita: ≥/< cohort-specific median. Educational level: ≥ High/≤ Middle school. Occupational class: class I-IV /V.

* Linear trend of cohort term a continuous scale, *P*<0.05.

(WHO z-score & 2-level models)

**Table S6.1.** Estimated difference (95%CI) in mean height between high and low SEP groups* at 7, 13, 17y for boys and girls in the earliest & latest cohorts†

|  | **Boy** |  | **Height** |  | **Girl** |  | **Height** |  |
| --- | --- | --- | --- | --- | --- | --- | --- | --- |
| **1981-85** | **Age(y)** | **high** | **low** | **high - low** |  | **high** | **low** | **high - low** |
| Urbanization Index | 7 | 118.4 (117.4, 119.4) | 116.5 (115.4, 117.6) | 1.9 (0.8, 3.1) |  | 116.6 (115.7, 117.5) | 113.6 (112.7, 114.6) | 3 (1.9, 4.1) |
|  | 13 | 151.1 (150.3, 151.8) | 146.7 (146, 147.5) | 4.3 (3.3, 5.3) |  | 149.7 (149, 150.4) | 146.4 (145.6, 147.1) | 3.3 (2.3, 4.3) |
|  | 17 | 169.3 (168.4, 170.1) | 166.4 (165.6, 167.3) | 2.8 (1.7, 3.9) |  | 159.2 (158.4, 160) | 157.5 (156.7, 158.3) | 1.7 (0.7, 2.8) |
| Household income/capita | 7 | 118.4 (117.4, 119.4) | 116.6 (115.5, 117.7) | 1.8 (0.7, 3) |  | 116.8 (116, 117.7) | 113.4 (112.5, 114.3) | 3.4 (2.3, 4.5) |
|  | 13 | 151 (150.3, 151.7) | 146.7 (145.9, 147.5) | 4.3 (3.3, 5.3) |  | 149.9 (149.2, 150.6) | 146.2 (145.5, 146.9) | 3.7 (2.7, 4.6) |
|  | 17 | 169.6 (168.8, 170.5) | 166.1 (165.3, 167) | 3.5 (2.4, 4.6) |  | 159.6 (158.8, 160.4) | 157.1 (156.3, 157.9) | 2.5 (1.4, 3.5) |
| Educational level | 7 | 119 (117.9, 120.2) | 116.8 (115.8, 117.8) | 2.2 (1, 3.5) |  | 116.6 (115.5, 117.7) | 114.6 (113.7, 115.4) | 2.1 (0.9, 3.3) |
|  | 13 | 151.3 (150.4, 152.2) | 147.6 (146.9, 148.3) | 3.7 (2.6, 4.8) |  | 149.8 (148.9, 150.7) | 147.2 (146.5, 147.8) | 2.6 (1.5, 3.7) |
|  | 17 | 169.6 (168.6, 170.7) | 167 (166.2, 167.8) | 2.7 (1.5, 3.9) |  | 159.5 (158.5, 160.5) | 157.8 (157.1, 158.6) | 1.7 (0.5, 2.8) |
| Occupational Class | 7 | 119 (117.8, 120.2) | 116.6 (115.7, 117.6) | 2.4 (1.1, 3.6) |  | 117 (115.9, 118.1) | 114.3 (113.4, 115.1) | 2.8 (1.6, 3.9) |
|  | 13 | 152.1 (151.2, 153) | 147.3 (146.6, 147.9) | 4.8 (3.7, 5.9) |  | 150.3 (149.4, 151.2) | 146.9 (146.3, 147.6) | 3.4 (2.3, 4.5) |
|  | 17 | 170.2 (169.2, 171.2) | 166.7 (165.9, 167.4) | 3.5 (2.4, 4.7) |  | 159.9 (159, 160.9) | 157.6 (156.8, 158.3) | 2.4 (1.2, 3.5) |
| **1996-2000** |  |  |  |  |  |  |  |  |
| Urbanization Index | 7 | 121.2 (120, 122.4) | 120.3 (119, 121.5) | 0.9 (-0.5, 2.4) |  | 121.2 (120, 122.4) | 117.3 (116.1, 118.5) | 3.9 (2.4, 5.3) |
|  | 13 | 156 (155.1, 157) | 152.7 (151.6, 153.7) | 3.4 (2, 4.7) |  | 154.7 (153.6, 155.7) | 150.5 (149.4, 151.5) | 4.2 (2.8, 5.6) |
|  | 17 | 172.8 (171.6, 174.1) | 171 (169.7, 172.3) | 1.8 (0.4, 3.2) |  | 162 (160.7, 163.2) | 159.3 (158.1, 160.6) | 2.6 (1.1, 4.1) |
| Household income/capita | 7 | 121.4 (120.2, 122.7) | 120.2 (119, 121.4) | 1.3 (-0.1, 2.6) |  | 121.6 (120.4, 122.8) | 117.2 (116, 118.3) | 4.5 (3.1, 5.9) |
|  | 13 | 156.2 (155.3, 157.2) | 152.5 (151.5, 153.5) | 3.7 (2.5, 5) |  | 155 (154, 156) | 150.3 (149.3, 151.3) | 4.7 (3.4, 6) |
|  | 17 | 173.3 (172.1, 174.5) | 170.4 (169.1, 171.7) | 2.9 (1.5, 4.3) |  | 162.5 (161.2, 163.7) | 159 (157.7, 160.3) | 3.5 (2.1, 4.9) |
| Educational level | 7 | 122.5 (121, 124) | 120.2 (119.1, 121.3) | 2.3 (0.7, 3.9) |  | 122.4 (120.9, 124) | 118.1 (117, 119.2) | 4.3 (2.7, 6) |
|  | 13 | 157 (155.7, 158.3) | 153.2 (152.3, 154.1) | 3.8 (2.3, 5.3) |  | 156.1 (154.7, 157.5) | 151.2 (150.3, 152.1) | 4.9 (3.3, 6.5) |
|  | 17 | 173.7 (172.1, 175.2) | 170.9 (169.7, 172.1) | 2.7 (1.2, 4.3) |  | 163.5 (161.9, 165.1) | 159.5 (158.4, 160.7) | 4 (2.3, 5.6) |
| Occupational Class | 7 | 121.9 (120.5, 123.3) | 120.2 (119, 121.4) | 1.7 (0.2, 3.2) |  | 121.3 (120, 122.7) | 117.7 (116.5, 118.9) | 3.6 (2.1, 5.2) |
|  | 13 | 156.9 (155.7, 158) | 152.8 (151.8, 153.7) | 4.1 (2.7, 5.5) |  | 155 (153.8, 156.1) | 150.7 (149.7, 151.7) | 4.3 (2.8, 5.7) |
|  | 17 | 173.6 (172.2, 175) | 170.7 (169.5, 172) | 2.9 (1.4, 4.3) |  | 162.4 (161, 163.8) | 159.2 (157.9, 160.4) | 3.2 (1.7, 4.8) |

Abbreviations: SEP: socio-economic position; CI: confidence interval.

* High/low SEP groups: urbanization Index and HH income per capita: ≥/< cohort-specific median. Educational level: ≥ High/≤ Middle school. Occupational class: class I-IV /V.

† All values were estimated from 2-level models (level-1: measurement; level-2: individual).

**Table S6.2.** Estimated difference (95%CI) in mean BMI between high and low SEP groups* at 7, 13, 17y for boys and girls in the earliest & latest cohorts†

|  | **Boy** |  | **BMI** |  | **Girl** |  | **BMI** |  |
| --- | --- | --- | --- | --- | --- | --- | --- | --- |
| **1981-85** | **Age(y)** | **high** | **low** | **high - low** |  | **high** | **low** | **high - low** |
| Urbanization Index | 7 | 15.5 (15.2, 15.7) | 15.1 (14.9, 15.4) | 0.4 (0, 0.7) |  | 14.8 (14.5, 15) | 14.7 (14.5, 15) | 0 (-0.3, 0.4) |
|  | 13 | 18 (17.8, 18.3) | 17.3 (17.1, 17.6) | 0.7 (0.4, 1.1) |  | 17.7 (17.5, 17.9) | 17.5 (17.3, 17.8) | 0.2 (-0.1, 0.5) |
|  | 17 | 20.2 (19.9, 20.5) | 19.8 (19.5, 20.2) | 0.3 (-0.1, 0.7) |  | 20 (19.7, 20.3) | 20.3 (20, 20.6) | -0.3 (-0.7, 0) |
| Household income/capita | 7 | 15.4 (15.2, 15.6) | 15.2 (14.9, 15.5) | 0.2 (-0.1, 0.6) |  | 14.8 (14.6, 15.1) | 14.7 (14.4, 14.9) | 0.2 (-0.2, 0.5) |
|  | 13 | 18 (17.7, 18.2) | 17.4 (17.2, 17.7) | 0.6 (0.2, 0.9) |  | 17.8 (17.6, 18.1) | 17.4 (17.2, 17.7) | 0.4 (0.1, 0.7) |
|  | 17 | 20.2 (19.9, 20.5) | 19.8 (19.5, 20.1) | 0.4 (0, 0.8) |  | 20.2 (19.9, 20.5) | 20.1 (19.8, 20.4) | 0.1 (-0.3, 0.4) |
| Educational level | 7 | 15.2 (14.9, 15.5) | 15.3 (15.1, 15.6) | -0.2 (-0.5, 0.2) |  | 14.7 (14.4, 15) | 14.8 (14.6, 15) | -0.1 (-0.5, 0.3) |
|  | 13 | 18 (17.7, 18.3) | 17.5 (17.3, 17.7) | 0.5 (0.1, 0.8) |  | 17.8 (17.5, 18) | 17.6 (17.4, 17.8) | 0.1 (-0.2, 0.5) |
|  | 17 | 20.3 (19.9, 20.6) | 19.9 (19.6, 20.2) | 0.3 (-0.1, 0.7) |  | 20 (19.6, 20.3) | 20.2 (20, 20.5) | -0.3 (-0.7, 0.1) |
| Occupational Class | 7 | 15.4 (15.1, 15.7) | 15.2 (15, 15.4) | 0.2 (-0.2, 0.5) |  | 14.9 (14.6, 15.2) | 14.7 (14.5, 14.9) | 0.2 (-0.2, 0.6) |
|  | 13 | 17.9 (17.7, 18.2) | 17.5 (17.3, 17.7) | 0.4 (0.1, 0.8) |  | 17.9 (17.7, 18.2) | 17.5 (17.3, 17.7) | 0.4 (0.1, 0.8) |
|  | 17 | 20.2 (19.8, 20.5) | 19.9 (19.6, 20.2) | 0.3 (-0.2, 0.7) |  | 20 (19.7, 20.4) | 20.2 (19.9, 20.5) | -0.2 (-0.6, 0.2) |
| **1996-2000** |  |  |  |  |  |  |  |  |
| Urbanization Index | 7 | 16.4 (16.1, 16.7) | 15.7 (15.4, 16) | 0.7 (0.3, 1.1) |  | 16.1 (15.8, 16.5) | 15.3 (14.9, 15.6) | 0.9 (0.4, 1.3) |
|  | 13 | 19 (18.7, 19.3) | 17.9 (17.6, 18.2) | 1 (0.6, 1.5) |  | 18.8 (18.5, 19.1) | 17.8 (17.5, 18.1) | 1 (0.6, 1.5) |
|  | 17 | 21.1 (20.6, 21.5) | 20.4 (20, 20.9) | 0.7 (0.2, 1.1) |  | 20.6 (20.1, 21.1) | 20.1 (19.6, 20.6) | 0.5 (0, 1) |
| Household income/capita | 7 | 16.6 (16.2, 16.9) | 15.6 (15.3, 15.9) | 1 (0.5, 1.4) |  | 16.1 (15.8, 16.4) | 15.3 (15, 15.6) | 0.8 (0.4, 1.2) |
|  | 13 | 19.1 (18.8, 19.4) | 17.8 (17.5, 18.1) | 1.3 (0.9, 1.7) |  | 18.8 (18.5, 19.2) | 17.8 (17.5, 18.1) | 1 (0.6, 1.4) |
|  | 17 | 21.3 (20.9, 21.8) | 20.2 (19.7, 20.7) | 1.1 (0.7, 1.6) |  | 20.7 (20.2, 21.2) | 20 (19.6, 20.5) | 0.7 (0.2, 1.2) |
| Educational level | 7 | 16.5 (16.1, 16.9) | 15.9 (15.7, 16.2) | 0.6 (0.1, 1) |  | 16.6 (16.2, 17.1) | 15.3 (15, 15.6) | 1.3 (0.8, 1.8) |
|  | 13 | 19.3 (18.9, 19.7) | 18.1 (17.8, 18.4) | 1.2 (0.7, 1.7) |  | 19.4 (19, 19.8) | 17.9 (17.6, 18.1) | 1.5 (1.1, 2) |
|  | 17 | 21.5 (21, 22.1) | 20.5 (20, 20.9) | 1.1 (0.6, 1.6) |  | 21.2 (20.6, 21.7) | 20 (19.6, 20.5) | 1.1 (0.6, 1.7) |
| Occupational Class | 7 | 16.5 (16.2, 16.9) | 15.7 (15.5, 16) | 0.8 (0.3, 1.2) |  | 16.3 (15.9, 16.7) | 15.2 (14.9, 15.5) | 1.1 (0.6, 1.5) |
|  | 13 | 19.1 (18.7, 19.5) | 18.1 (17.8, 18.4) | 1 (0.6, 1.5) |  | 19.1 (18.7, 19.4) | 17.8 (17.5, 18.1) | 1.3 (0.8, 1.7) |
|  | 17 | 21.4 (20.9, 21.9) | 20.5 (20, 21) | 0.9 (0.4, 1.4) |  | 20.7 (20.2, 21.2) | 20 (19.5, 20.5) | 0.7 (0.2, 1.2) |

Abbreviations: SEP: socio-economic position; CI: confidence interval.

* High/low SEP groups: urbanization Index and HH income per capita: ≥/< cohort-specific median. Educational level: ≥ High/≤ Middle school. Occupational class: class I-IV /V.

† All values were estimated from 2-level models (level-1: measurement; level-2: individual).

**Table S7:** Estimated difference (95%CI) in mean height and BMI between high and low SEP groups* at 10y for the earliest & latest cohorts†

|  |  |  | **Height** |  |  | **BMI** |  |
| --- | --- | --- | --- | --- | --- | --- | --- |
| **Boys** | **Cohort** | **High** | **Low** | **High- low** | **High** | **Low** | **High- low** |
| Urbanization Index | 1981-85 | 132.3 (131.6, 133) | 128.5 (127.8, 129.2) | 3.8 (2.9, 4.8) | 16.3 (16.1, 16.5) | 15.8 (15.6, 16) | 0.5 (0.2, 0.8) |
|  | 1986-90 | 134.1 (133.4, 134.8) | 130.3 (129.7, 131) | 3.8 (2.9, 4.7) | 16.7 (16.4, 16.9) | 16.2 (16, 16.4) | 0.5 (0.2, 0.8) |
|  | 1991-95 | 135.3 (134.4, 136.3) | 132.1 (131.2, 133) | 3.2 (2, 4.5) | 17 (16.6, 17.3) | 16.1 (15.9, 16.4) | 0.8 (0.4, 1.2) |
|  | 1996-2000 | 136.8 (135.9, 137.8) | 134 (133, 134.9) | 2.9 (1.6, 4.1) | 17.2 (16.9, 17.5) | 16.4 (16.1, 16.7) | 0.8 (0.4, 1.3) |
| HH income/ capita | 1981-85 | 132.2 (131.5, 132.9) | 128.5 (127.8, 129.3) | 3.6 (2.6, 4.6) | 16.2 (16, 16.4) | 15.9 (15.6, 16.1) | 0.4 (0, 0.7) |
|  | 1986-90 | 134 (133.3, 134.6) | 130.3 (129.6, 131) | 3.6 (2.7, 4.5) | 16.5 (16.3, 16.7) | 16.3 (16.1, 16.5) | 0.2 (-0.1, 0.5) |
|  | 1991-95 | 134.8 (133.8, 135.7) | 132.4 (131.5, 133.4) | 2.3 (1.1, 3.6) | 16.9 (16.6, 17.2) | 16.1 (15.8, 16.4) | 0.8 (0.4, 1.2) |
|  | 1996-2000 | 137 (136, 137.9) | 133.9 (133, 134.8) | 3.1 (1.8, 4.3) | 17.4 (17.1, 17.7) | 16.3 (16, 16.5) | 1.1 (0.7, 1.5) |
| Educational level | 1981-85 | 132.6 (131.8, 133.5) | 129.2 (128.6, 129.8) | 3.4 (2.4, 4.5) | 16.1 (15.8, 16.3) | 16 (15.8, 16.2) | 0.1 (-0.3, 0.4) |
|  | 1986-90 | 134 (133.2, 134.8) | 131 (130.4, 131.7) | 2.9 (2, 3.9) | 16.5 (16.3, 16.8) | 16.3 (16.2, 16.5) | 0.2 (-0.1, 0.5) |
|  | 1991-95 | 135.6 (134.4, 136.8) | 132.7 (131.8, 133.5) | 3 (1.6, 4.4) | 16.8 (16.4, 17.2) | 16.4 (16.1, 16.6) | 0.4 (0, 0.9) |
|  | 1996-2000 | 137.9 (136.7, 139.1) | 134.4 (133.6, 135.2) | 3.5 (2.1, 4.9) | 17.4 (17, 17.8) | 16.6 (16.3, 16.8) | 0.8 (0.4, 1.3) |
| Occupational Class | 1981-85 | 133.2 (132.3, 134.1) | 129 (128.3, 129.6) | 4.2 (3.2, 5.3) | 16.2 (15.9, 16.5) | 15.9 (15.7, 16.1) | 0.3 (-0.1, 0.6) |
|  | 1986-90 | 134.7 (133.9, 135.5) | 130.8 (130.2, 131.4) | 3.9 (2.9, 4.9) | 16.9 (16.6, 17.1) | 16.2 (16, 16.4) | 0.7 (0.4, 1) |
|  | 1991-95 | 135.7 (134.6, 136.9) | 132.4 (131.6, 133.3) | 3.3 (2, 4.7) | 16.7 (16.3, 17) | 16.4 (16.1, 16.6) | 0.3 (-0.2, 0.7) |
|  | 1996-2000 | 137.6 (136.6, 138.7) | 134.1 (133.2, 135) | 3.5 (2.2, 4.9) | 17.3 (17, 17.7) | 16.5 (16.2, 16.7) | 0.9 (0.4, 1.3) |
| **Girls** |  |  |  |  |  |  |  |
| Urbanization Index | 1981-85 | 132.9 (132.2, 133.6) | 129.5 (128.8, 130.3) | 3.4 (2.4, 4.3) | 15.7 (15.5, 15.9) | 15.6 (15.4, 15.8) | 0.1 (-0.2, 0.4) |
|  | 1986-90 | 135.2 (134.5, 135.9) | 131.2 (130.5, 131.9) | 4 (3, 4.9) | 16.2 (16, 16.4) | 15.8 (15.6, 16) | 0.4 (0.1, 0.7) |
|  | 1991-95 | 136.1 (135.2, 137.1) | 133.5 (132.6, 134.5) | 2.6 (1.3, 3.8) | 16.4 (16.1, 16.7) | 15.9 (15.6, 16.2) | 0.5 (0.1, 0.8) |
|  | 1996-2000 | 137.9 (136.9, 139) | 133.7 (132.7, 134.7) | 4.2 (2.9, 5.6) | 17 (16.7, 17.3) | 16.1 (15.8, 16.4) | 1 (0.6, 1.4) |
| HH income/ capita | 1981-85 | 133.1 (132.4, 133.8) | 129.4 (128.6, 130.1) | 3.7 (2.7, 4.7) | 15.8 (15.6, 16) | 15.5 (15.3, 15.7) | 0.3 (0, 0.6) |
|  | 1986-90 | 134.9 (134.2, 135.6) | 131.3 (130.6, 132) | 3.6 (2.7, 4.6) | 16 (15.8, 16.2) | 16 (15.8, 16.2) | 0 (-0.3, 0.3) |
|  | 1991-95 | 136.9 (135.9, 137.8) | 133.1 (132.2, 134) | 3.8 (2.6, 5) | 16.4 (16.1, 16.6) | 15.9 (15.7, 16.2) | 0.4 (0, 0.8) |
|  | 1996-2000 | 138.3 (137.3, 139.3) | 133.5 (132.6, 134.5) | 4.7 (3.4, 6) | 17 (16.7, 17.3) | 16.1 (15.8, 16.4) | 0.9 (0.5, 1.3) |
| Educational level | 1981-85 | 132.9 (132, 133.8) | 130.4 (129.8, 131.1) | 2.4 (1.4, 3.5) | 15.7 (15.4, 16) | 15.7 (15.5, 15.9) | 0 (-0.3, 0.4) |
|  | 1986-90 | 135.3 (134.5, 136.2) | 132 (131.4, 132.6) | 3.3 (2.3, 4.4) | 16 (15.8, 16.3) | 15.9 (15.8, 16.1) | 0.1 (-0.2, 0.4) |
|  | 1991-95 | 138.2 (136.9, 139.4) | 133.5 (132.7, 134.3) | 4.7 (3.3, 6.1) | 16.6 (16.2, 16.9) | 16 (15.8, 16.2) | 0.5 (0.1, 1) |
|  | 1996-2000 | 139.2 (137.8, 140.6) | 134.5 (133.6, 135.4) | 4.7 (3.1, 6.3) | 17.6 (17.2, 18) | 16.1 (15.9, 16.4) | 1.4 (0.9, 1.9) |
| Occupational Class | 1981-85 | 133.4 (132.5, 134.3) | 130.2 (129.5, 130.8) | 3.2 (2.1, 4.3) | 15.9 (15.6, 16.2) | 15.6 (15.4, 15.8) | 0.3 (0, 0.7) |
|  | 1986-90 | 136 (135.1, 136.8) | 131.7 (131.1, 132.3) | 4.3 (3.2, 5.3) | 16.3 (16, 16.5) | 15.8 (15.6, 16) | 0.5 (0.2, 0.8) |
|  | 1991-95 | 137.5 (136.4, 138.6) | 133.3 (132.5, 134.2) | 4.2 (2.9, 5.5) | 16.7 (16.4, 17.1) | 15.8 (15.5, 16) | 0.9 (0.5, 1.3) |
|  | 1996-2000 | 138.1 (137, 139.3) | 134 (133.1, 135) | 4.1 (2.6, 5.5) | - 1. (16.9, 17.6) | 16 (15.8, 16.3) | 1.2 (0.8, 1.6) |

* High/low SEP groups: urbanization Index and HH income per capita: ≥/< cohort-specific median. Educational level: ≥ High/≤ Middle school. Occupational class: class I-IV /V.

**Table S8.** Estimated prevalence (95%CI) of stunting, thinness and overweight ^§^ by SEP groups * and their ORs (high vs low SEP) across cohorts †

| **Cohort** | **Stunting** | | | **Thinness** | | | **Overweight** | | |
| --- | --- | --- | --- | --- | --- | --- | --- | --- | --- |
|  | **High** | **Low** | **OR (95% CI)** | **High** | **Low** | **OR (95% CI)** | **High** | **Low** | **OR (95% CI)** |
| **Urbanization Index** | **≥ median** | **< median** |  | **≥ median** | **< median** |  | **≥ median** | **< median** |  |
| 1981-85 | 15.0% | 28.5% | 0.44 (0.35, 0.56) | 6.3% | 6.8% | 0.92 (0.69, 1.23) | 10.9% | 6.4% | 1.77 (1.3, 2.41) |
| 1986-90 | 10.6% | 20.6% | 0.46 (0.36, 0.58) | 6.7% | 7.2% | 0.92 (0.7, 1.21) | 14.9% | 9.9% | 1.6 (1.23, 2.08) |
| 1991-95 | 9.5% | 13.4% | 0.68 (0.47, 0.97) | 5.5% | 6.0% | 0.91 (0.61, 1.36) | 17.8% | 10.9% | 1.77 (1.26, 2.5) |
| 1996-2000 | 6.0% | 12.6% | 0.44 (0.3, 0.66) | 6.1% | 8.5% | 0.7 (0.48, 1.04) | 27.4% | 15.2% | 2.1 (1.53, 2.89) |
| Total | 10.8% | 19.9% | 0.49 (0.42, 0.56) | 6.3% | 7.1% | 0.88 (0.75, 1.03) | 16.2% | 9.9% | 1.77 (1.52, 2.06) |
| **HH Income/capita** | **≥ median** | **< median** |  | **≥ median** | **< median** |  | **≥ median** | **< median** |  |
| 1981-85 | 13.4% | 31.5% | 0.34 (0.27, 0.42) | 5.9% | 7.2% | 0.81 (0.61, 1.09) | 10.4% | 6.8% | 1.59 (1.17, 2.16) |
| 1986-90 | 10.5% | 21.6% | 0.42 (0.33, 0.54) | 7.1% | 6.8% | 1.06 (0.8, 1.4) | 13.3% | 11.0% | 1.24 (0.96, 1.61) |
| 1991-95 | 7.8% | 15.5% | 0.46 (0.32, 0.67) | 4.5% | 6.9% | 0.64 (0.42, 0.96) | 17.6% | 10.5% | 1.82 (1.29, 2.57) |
| 1996-2000 | 6.1% | 12.7% | 0.44 (0.3, 0.66) | 6.2% | 8.4% | 0.72 (0.49, 1.07) | 28.8% | 14.1% | 2.46 (1.79, 3.39) |
| Total | 10.0% | 21.5% | 0.41 (0.35, 0.47) | 6.2% | 7.2% | 0.84 (0.72, 0.99) | 15.6% | 10.1% | 1.64 (1.41, 1.9) |
| **Educational Level** | **≥ High school** | **<high school** |  | **≥ High school** | **<high school** |  | **≥ High school** | **<high school** |  |
| 1981-85 | 13.1% | 26.1% | 0.42 (0.33, 0.55) | 7.2% | 6.1% | 1.2 (0.89, 1.63) | 10.4% | 7.3% | 1.48 (1.08, 2.03) |
| 1986-90 | 10.8% | 18.6% | 0.53 (0.41, 0.69) | 7.5% | 6.6% | 1.14 (0.85, 1.52) | 14.2% | 11.1% | 1.33 (1.01, 1.74) |
| 1991-95 | 5.7% | 14.2% | 0.36 (0.23, 0.57) | 4.2% | 6.4% | 0.64 (0.4, 1.03) | 17.0% | 12.6% | 1.43 (1, 2.04) |
| 1996-2000 | 5.1% | 11.2% | 0.42 (0.26, 0.7) | 4.1% | 8.4% | 0.46 (0.28, 0.77) | 32.9% | 16.6% | 2.46 (1.76, 3.44) |
| Total | 9.7% | 18.4% | 0.47 (0.4, 0.56) | 6.4% | 6.8% | 0.94 (0.79, 1.12) | 16.1% | 11.1% | 1.53 (1.31, 1.78) |
| **Occupational Class** | **Classes I-IV** | **Class V** |  | **Classes I-IV** | **Class V** |  | **Classes I-IV** | **Class V** |  |
| 1981-85 | 11.9% | 26.2% | 0.38 (0.29, 0.5) | 5.5% | 6.9% | 0.79 (0.57, 1.09) | 10.8% | 7.3% | 1.54 (1.12, 2.11) |
| 1986-90 | 10.0% | 18.7% | 0.49 (0.37, 0.64) | 5.5% | 7.6% | 0.71 (0.52, 0.97) | 16.3% | 10.0% | 1.75 (1.34, 2.29) |
| 1991-95 | 6.5% | 14.4% | 0.41 (0.27, 0.63) | 4.3% | 6.5% | 0.65 (0.42, 1.02) | 16.7% | 12.1% | 1.46 (1.02, 2.07) |
| 1996-2000 | 5.6% | 12.0% | 0.44 (0.28, 0.68) | 5.5% | 8.2% | 0.65 (0.43, 1) | 30.1% | 15.4% | 2.37 (1.71, 3.27) |
| Total | 8.9% | 19.0% | 0.42 (0.35, 0.49) | 5.3% | 7.3% | 0.71 (0.59, 0.86) | 17.3% | 10.3% | 1.83 (1.56, 2.13) |

Abbreviations: HH: household; SEP: socio-economic position; OR: odds ratio; CI: confidence interval.

§ Stunting, thinness and overweight were defined by WHO 2007 reference.

* High/low SEP groups: urbanization Index and HH income per capita: ≥/< cohort-specific median. Educational level: ≥ High/≤ Middle school. Occupational class: class I-IV /V.

† All values estimated for **boys aged 10y** from 2-level (level-1: measurement; level-2: individual) logistic models with adjustment for age and sex.
